# Tet protein function during Drosophila development

**DOI:** 10.1371/journal.pone.0190367

**Published:** 2018-01-11

**Authors:** Fei Wang, Svetlana Minakhina, Hiep Tran, Neha Changela, Joseph Kramer, Ruth Steward

**Affiliations:** 1 Rutgers University, Department of Molecular Biology, Waksman Institute, Piscataway, NJ United States of America; 2 Department of Pathology and Laboratory Medicine, Rutgers-Robert Wood Johnson Medical School; Rutgers, The State University of New Jersey, New Brunswick, NJ, United States of America; 3 Cancer Institute of New Jersey, New Brunswick, NJ, United States of America; Inc, UNITED STATES

## Abstract

The TET (Ten-eleven translocation) 1, 2 and 3 proteins have been shown to function as DNA hydroxymethylases in vertebrates and their requirements have been documented extensively. Recently, the Tet proteins have been shown to also hydroxylate 5-methylcytosine in RNA. 5-hydroxymethylcytosine (5hmrC) is enriched in messenger RNA but the function of this modification has yet to be elucidated. Because Cytosine methylation in DNA is barely detectable in Drosophila, it serves as an ideal model to study the biological function of 5hmrC. Here, we characterized the temporal and spatial expression and requirement of Tet throughout Drosophila development. We show that Tet is essential for viability as Tet complete loss-of-function animals die at the late pupal stage. Tet is highly expressed in neuronal tissues and at more moderate levels in somatic muscle precursors in embryos and larvae. Depletion of Tet in muscle precursors at early embryonic stages leads to defects in larval locomotion and late pupal lethality. Although Tet knock-down in neuronal tissue does not cause lethality, it is essential for neuronal function during development through its affects upon locomotion in larvae and the circadian rhythm of adult flies. Further, we report the function of Tet in ovarian morphogenesis. Together, our findings provide basic insights into the biological function of Tet in Drosophila, and may illuminate observed neuronal and muscle phenotypes observed in vertebrates.

## Introduction

RNA modifications represent a newly discovered layer of epigenetic regulation with great importance in development [1]. RNA contains more than 100 distinct modifications, most of which are in abundant noncoding RNAs [2]. N6-methyladenosine (m6A) is the most abundant modification in mRNAs. It plays an important role in neuronal function, and in Drosophila it is also required for sex determination [3]. We have recently demonstrated the presence of an additional RNA modification, 5-hydroxymethylcytosine (5hmC) on mRNA, which is regulated by the Drosophila Tet protein [4].

In vertebrates, the TET proteins (Ten-eleven translocation) were identified as DNA modification enzymes. They function as oxoglutarate- and iron-dependent dioxygenases that oxidize 5-methylcytosine (5mC) to 5-hydroxy-methylcytosine (5hmC) ([5–8]. 5hmC is versatile; it can serve as an epigenetic mark itself, or it can serve as an intermediate leading to the TET-dependent removal of the methyl mark through a series of intermediates which themselves serve as epigenetic marks[9, 10]. It has been shown that 5hmC is present in gene bodies, where the modification positively correlates with gene expression levels [11]. Gain of 5hmC is usually correlated with the loss of H3K27me3 and activation of gene expression [12]. In mammals, the Tet family contains three members, Tet1, Tet2 and Tet3, that share a high degree of homology within their C-terminal catalytic domain. Tet1 and Tet3 also share a CXXC patterned DNA binding domain. The regulation of gene transcription by TETs is complicated and still needs to be elucidated, especially in view of the new role in RNA hydroxymethylation. Tet1 and Tet2 are highly expressed in mouse embryonic stem cells (ESCs). ESCs depleted for both Tet1 and Tet2 retain pluripotency but show reduced levels of 5hmC in DNA, but complete loss of either Tet1 or Tet2 does not affect viability [13, 14]. Tet3 homozygous mutant mouse embryos develop normally, but die at birth. This is apparently due to a defect in epigenetic reprogramming during zygotic development [8]. TET3 has been shown to catalyze the conversion of 5mC to 5hmC in RNA derived from tissue culture cells and mouse ESCs, similar to what we have reported in Drosophila [4, 15].

In Drosophila, DNA methylation is only observed in early embryonic stages at barely detectable levels and is not believed to have a function in genome-wide regulation of gene expression[16, 17]. The fly genome does not encode any canonical DNA methyltransferase 1 or 3 (Dnmt1/3) homologs. Dnmt2 is the only known candidate DNA methyltransferase in Drosophila and has been demonstrated to modify specific t-RNAs. Dnmt2 null mutants are homozygous viable, and whole-genome bisulfite sequencing did not uncover Dnmt-dependent cytosine methylation patterns in Drosophila [18, 19]. The enzyme(s) that control cytosine methylation (5mrC) in Drosophila mRNA are not known.

The fly genome contains one conserved Tet gene. Previously, we have shown that the 5hmrC modification is found in Drosophila polyA^+^ RNA and that upon Tet knock down (KD) in S2 Schneider cells, the level of 5hmrC is reduced by 50%. *Tet* is essential, as null mutants are lethal at the late pupal stage. 5hmrC is enriched in neuronal tissues and is highest in RNA isolated from 3^rd^ instar larval brains. 5hmrC was also reduced significantly in *Tet*^*null*^ brains. Further, we mapped the transcriptome-wide distribution of RNA 5hmC in Drosophila S2 cells and found that the modification targets specific transcripts. 5hmrC modified mRNA is found in association with polysomes, suggesting that this modification facilitates mRNA translation [4]. The almost complete absence of 5mC and 5hmC on DNA makes Drosophila an excellence platform to investigate the importance of RNA 5hmC (4).

Here we characterize the expression pattern of Tet and its functional requirement. Tet is expressed most highly in neuronal cells during development and at lower levels in muscle precursors during embryogenesis as well as in the larval imaginal discs. Tissue-specific and temporally controlled knock-down indicates that Tet function is required during embryogenesis for larval locomotion, ovary development, and during embryonic or larval stages in PDF neurons to control aspects of the adult circadian rhythm. Our identification of Tet requirements in Drosophila establishes its importance of 5hmrC for muscle and neuronal function.

## Results

### The Tet protein and its functional domains

**Fig 1:**
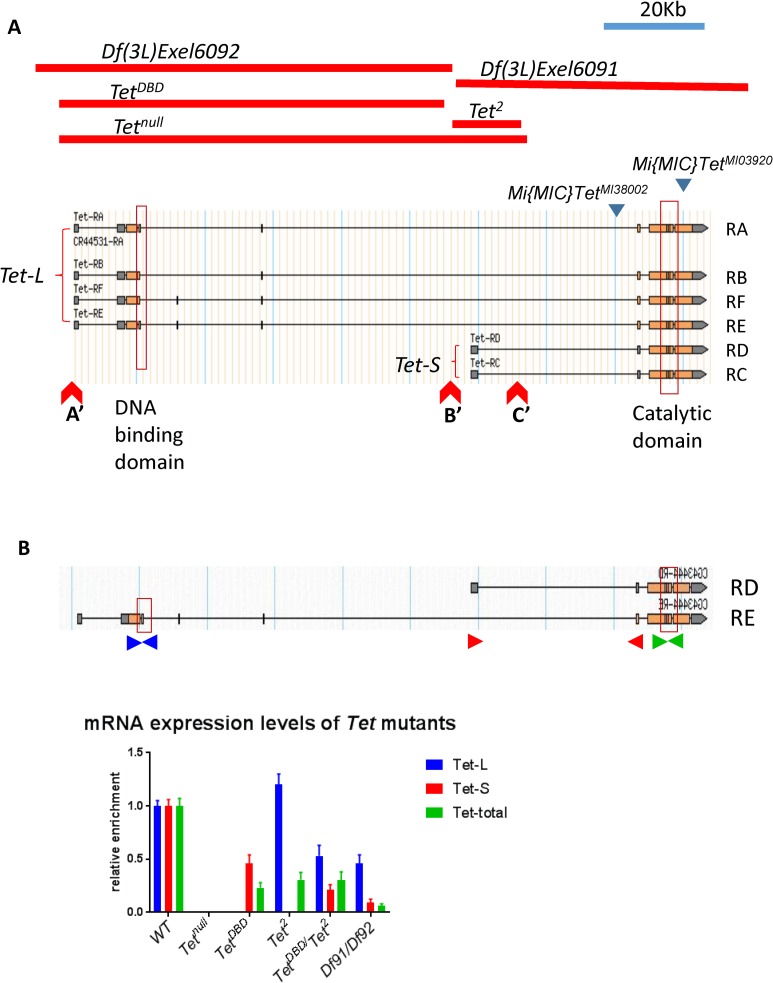
The Tet locus. (A) The different Tet transcripts (Tet-L and Tet-S) and the location of the DNA-binding and catalytic domains (boxes), as well as the two Mi{MIC} insertions (blue triangles) are indicated. Red lines show the extent of the *Tet* deletions. Red Triangles mark insertion sites of the piggyback transposons used for induction of deletions. (B) The *Tet-L* and *Tet-*S transcripts and their expression levels in WT and *Tet* brains. Primer pairs specific to the long transcript (TetL, blue), the short transcript (TetS, red) and all transcripts (green) were used. Df91/Df92 = *Df(3L)Exel6091*/*Df(3L)Exel609*. qRT-PCR results are normalized to *Rp49 (RpL32)*.

The Drosophila genome contains a single *Tet* gene encoding 6 transcripts that together appear to encompass transcripts encoded by the three vertebrate genes. Two Drosophila transcripts encode only the catalytic domain, similar to the transcripts of the vertebrate Tet2 gene (*Tet-S*, Fig 1A). Similar to the gene products of vertebrate Tet1 and 3, the other four transcripts are larger and encode in addition to the catalytic domain the CXXC DNA binding domain (*Tet-L*, Fig 1A). The domain organization is maintained in the vertebrate and Drosophila proteins and the amino acid similarity is highly conserved in the active domains [20]. When we initiated this work, two deletions, *Df(3L)Exel6091* and *Df(3L)Exel6092*, that originate from the same site within the Tet coding region and extend in opposite directions were available (Fig 1A). In *trans*, these deficiencies were mostly lethal at the late pupal stage with ~ 20% escapers that lived only for a few days, indicating that the *Tet* gene has an essential function. To further determine the importance of *Tet* we created a null allele that deletes the 5’ end of all the transcripts. Homozygous *Tet*^*null*^ animals develop up to late pupal-pharate adult stages, but no flies eclose [4].

To investigate the functional requirements for the two different forms of Tet, we aimed at inducing transcript-specific deletions within the Tet gen. Because several fly lines carrying piggyBac transposons at suitable sites in the Tet gene were available we used FRT-mediated site-specific recombination of these transposons (A’ to C’ in Fig 1A) that allowed us to isolate targeted deletions. Recombination between A’ and B’ resulted in the deletion of the 5’ end of Tet-L including the DNA binding domain (*Tet*^*DBD*^). Recombination between B’ and C’ caused the deletion of the 5’ end of Tet-S (*Tet*^*2*^). Recombination between A’ and C’ caused the deletion of the 5’ end of both isoforms (*Tet*^*null*^). To confirm the effect of these deletions on the Tet transcripts, q-PCR was performed with RNA extracted from 3^rd^ instar larval brains using transcript specific primers (Fig 1B). *Tet*^*null*^ mutants have neither Tet-L nor Tet-S transcripts. *Tet*^*DBD*^ mutants do not produce any long transcripts but the short transcripts are present at about 50% of the level observed in WT. With the primers common to all transcripts, we observe 25% of total (Tet-L and Tet-S) transcripts (Fig 1B). *Tet*^*2*^ mutants show no short transcripts, but, while more than normal levels of fragments were amplified with the N-terminal primers, the C-terminal primers suggest that only about 50% of the transcripts were full-length (Fig 1B). *Tet*^*DBD*^/*Tet*^*2*^ transheterozygotes and *Df(3L)Exel6091*/ *Df(3L)Exel6092* show similar results. About 50% of Tet-L and 5–10% of Tet-S, or 5–25% of full length RNA can be detected (Fig 1B).

Despite the presence of varying levels of Tet transcripts, *Tet*^*DBD*^ and *Tet*^*2*^ over *Tet*^*null*^ or as homzygotes are lethal at the late pupae-pharate adult stage as *Tet*^*null*^. The transheterozygous *Tet*^*DBD*^/*Tet*^*2*^ animals show a 100% eclosion rate and the flies live for more than 2 weeks (Table 1), but they are sterile and have normal looking ovaries, full of eggs that are not laid (S5C Fig). The *Df(3L)Exel6091*/ *Df(3L)Exel6092* heterozygotes show a stronger phenotype than *Tet*^*DBD*^/*Tet*^*2*^; about 20% of flies eclose, but die within one or two days, reflecting the lower level of Tet expression.

**Table 1:** The *Tet* alleles.

Genotype	Survival stage	Adult survival rate
*Df(3L)Exel6091/ Df(3L)Exel6092*	Late pupa	20% (escapers)
*Tet*^*null*^	Late Pupa	0
*Tet*^*DBD*^	Late pupa	0
*Tet*^*DBD*^*/ Tet*^*null*^	Late pupa	0
*Tet*^*2*^	Late pupa	0
Tet^2^ / Tet^null^	Late pupa	0
Tet^DBD^/Tet^2^	Adults	100%
*Mi{MIC}Tet*^*MI03920*^*/Tet*^*null*^	Adults with severe moving disability	100%

We obtained an additional Tet mutant line, *Mi{MIC}Tet*^*MI03920*^, carrying the Mi{MIC} insertion in the last exon of the *Tet* gene (Fig 1A). *Mi{MIC}Tet*^*MI03920*^ shows 100% eclosion rate over *Tet*^*null*^. The flies have extended wings (S5A Fig), exhibit strong defects in coordinated motor control and die within 1–2 days after eclosion (Table 1). They also exhibit highly abnormal ovaries (S5B Fig). This indicates that *Mi{MIC}Tet*^*MI03920*^ *is* a hypomorphic allele.

### Tet is most highly expressed in embryonic neurons and also in muscle precursors

**Fig 2:**
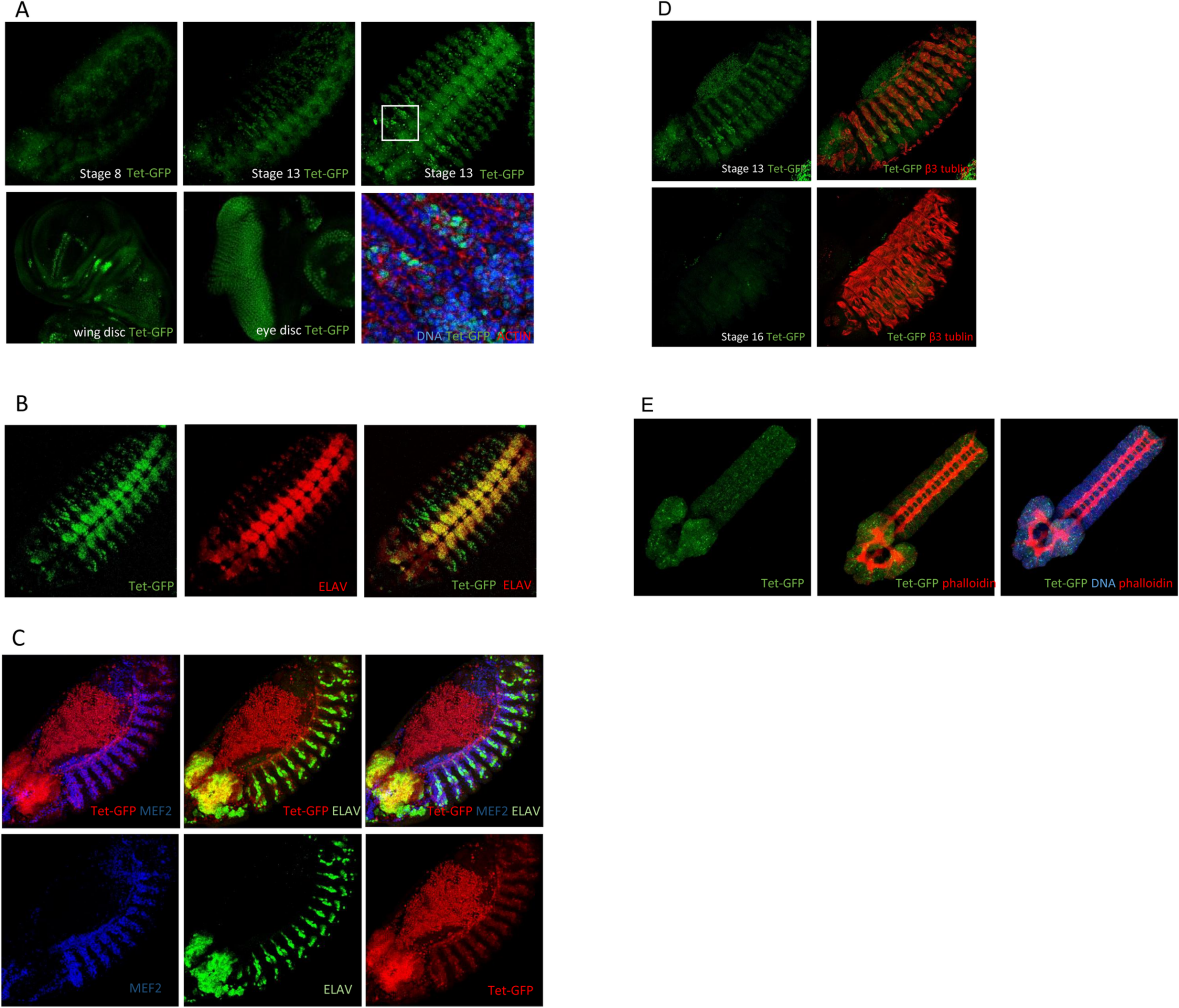
Tet expression. (A) Native Tet-GFP is detected first at the germ band extension stage (side view) and becomes more pronounced in neuronal cells by stage 13 (side view). At stage 14 (ventral view) the protein is clearly nuclear (see enlargement below) The protein is also detected most strongly in proneural cells of the wing disc and in the developing photoreceptors behind the morphogenetic furrow. (B) Tet-GFP and the neuronal marker Elav overlap in the central nervous system. Stage 14 embryo, ventral view. (C) Tet expression shows significant overlap with the mesodermal marker Mef2, but it its expression in mesodermal cells is lower than in neuronal cells. Stage 13 embryo; note the autofluorescence of the yolk mass on the dorsal side of the embryo. (D) Tet-GFP is expressed in differentiating mesodermal cells, labeled by beta 3 tubulin in stage 13 embryos; by stage 16 Tet-GFP is no longer detected. (E) Central nervous system dissected from 1^st^ instar larva (24h). Tet-GFP is expressed in the central nervous system. throughout embryogenesis. Phalloidin stain represents F-actin. NB: all figures show endogenous Tet-GFP expression, except for B which is stained with anti-GFP antibody.

Two Mi(MIC) insertion lines are available. One of them, mentioned above, contains the insert in the last exon and results in a mutant phenotype. The second one, *Mi{MIC}Tet*^*MI05009*^, does no show a phenotype and has the insert in an intronic site, common to both Tet-L and Tet-S, just in front of the second last exon. Through cassette exchange with the Mi{MIC} insertion we generated a line expressing a Tet-GFP fusion protein under the control of the endogenous Tet promoters, *Tet-GFP* (called *dTet-Mi* in [4]). *Tet-GFP* shows no phenotype over *Tet*^*null*^, and is homozygous viable, indicating that the GFP-Tet fusion proteins are functional. We find that Tet-GFP is not maternally contributed and is first detected at the germ band extension stage, mainly in the central nervous system (Fig 2A, S1B Fig), consistent with *Tet* mRNA expression (S1A Fig). Its expression overlaps largely with the postmitotic neuronal marker, ELAV (Fig 2B and 2C). Tet is also expressed, albeit at lower levels, in the somatic muscle precursors as they fuse into myotubes and migrate towards the dorsal aspect of the embryo. Tet is enriched in nuclei, and its expression overlaps with the nuclear mesodermal cell marker Mef2 (Fig 2C). Tet-GFP expression subsides in muscle cells at stage 16 shortly before hatching (Fig 2D), but the expression in the central nervous system is continuously present (Fig 2E). This temporal expression during embryogenesis is consistent with *Tet* RNA expression described previously (S1 Fig) [4].

In addition to the CNS, Tet-GFP is present in various imaginal discs. In wing discs, Tet expression is strongest in the proneural clusters [21] and lower in myoblasts that develop into direct flight muscles in adults (Fig 2A). In the eye discs, Tet expresses in all neuronal cells of the developing photoreceptor cells posterior to the morphogenetic furrow (Fig 2A). Taken together our results show that Tet is most highly expressed in the neuronal lineages throughout development and is also present in the somatic muscle precursors. This expression pattern is consistent with our observation that Tet is required in neural and muscle cells.

### Tissue-specific and stage-specific requirement of *Tet*

To further address the tissue-specific requirement of Tet, we depleted Tet in specific tissues and cell types using the UAS-Gal4 system. We have obtained four different *Tet*-RNAi lines (VDRC) [22]. We first tested the knock-down (KD) efficiency of these different RNAi lines in RNA from 0-12h embryos.

The RNAi expression was driven by the general *da-gal4* driver. RT-PCR was performed to estimate the mRNA KD. One of the lines (#v102273, we call *Tet*^*RNAi*^) was selected for future experiments because we observed ~85% KD in mRNA levels (S2A and S2B Fig) and the animals die at late pupal stages similar to *Tet* null mutants. The other lines showed no lethality and more moderate KD of Tet mRNA, around 50%-70% (S2A Fig). When *UAS-Tet*^*RNAi*^ was combined with other ubiquitous drivers, such as tub-gal4 or act5c-gal4, the animals also died before eclosion (Table 2). The broadly expressed mesodermal drivers mef2-gal4 or how24B-gal4 driving *Tet*^*RNAi*^ also cause lethality similar to that observed in null mutants. Other muscle drivers that express at later stages, such as G14-gal4 and act88F-gal4, do not cause lethality (see Table 2). This suggests that the requirement of Tet in muscle development occurs during early stages of development. Notably, Tet KD under the control of several neuron drivers (Table 2), did not result in lethality. This indicates that Tet is essential for the function of muscles, but does not rule out that Tet could also affect neuronal functions.

**Table 2:** Phenotypes of Tet KD with different drivers.

Driver name	Expression pattern	Lethality with Tet RNAi
da-gal4	Ubiquitous; maternal	Just before eclosion
act5C-gal4	Ubiquitous expression with an early onset	Just before eclosion
Mef2-gal4	Mesoderm, embryonic stage 12 myoblast and larval muscles	Just before eclosion
how24B-gal4	Mesoderm and larval muscles	Just before eclosion with dcr2 (viable otherwise)
G14-gal4	Muscle cells from stage 11 to larvae (2,3,4,5,6)	viable
act88F-gal4	Wing flight muscle	viable
hand-gal4	Cardiac lineages	viable
tin-gal4	heart	viable
elav-gal4	All postmitotic neurons with an early onset	viable with dcr2
OK6-gal4	all motor neurons, salivary glands, wing discs and a subset of tracheal branches commencing the first instar larval stage and persisting until pupation	viable
nrv2-gal4	Nervous system-specific expression from embryo to the adult stage	viable

### *Tet* is required for locomotion in neurons and muscle precursors

We examined the *Tet*^*null*^ embryonic nervous systems and 3^rd^ instar larval brains as compared to WT using cell-type specific markers and found no substantial difference in organization or morphology. This lead us to investigate potential functional defects in the nervous system. Locomotion behaviors are coordinated by neurons, muscles, and neuromuscular junctions. Given the Tet expression pattern and the effects of tissue specific knockdown it seemed a possible biological endpoint to assess. Therefore, we set up tests to study the locomotion of third instar larvae. The external morphology of WT and mutant 3^rd^ instar larvae is indistinguishable (S3A Fig).

**Fig 3:**
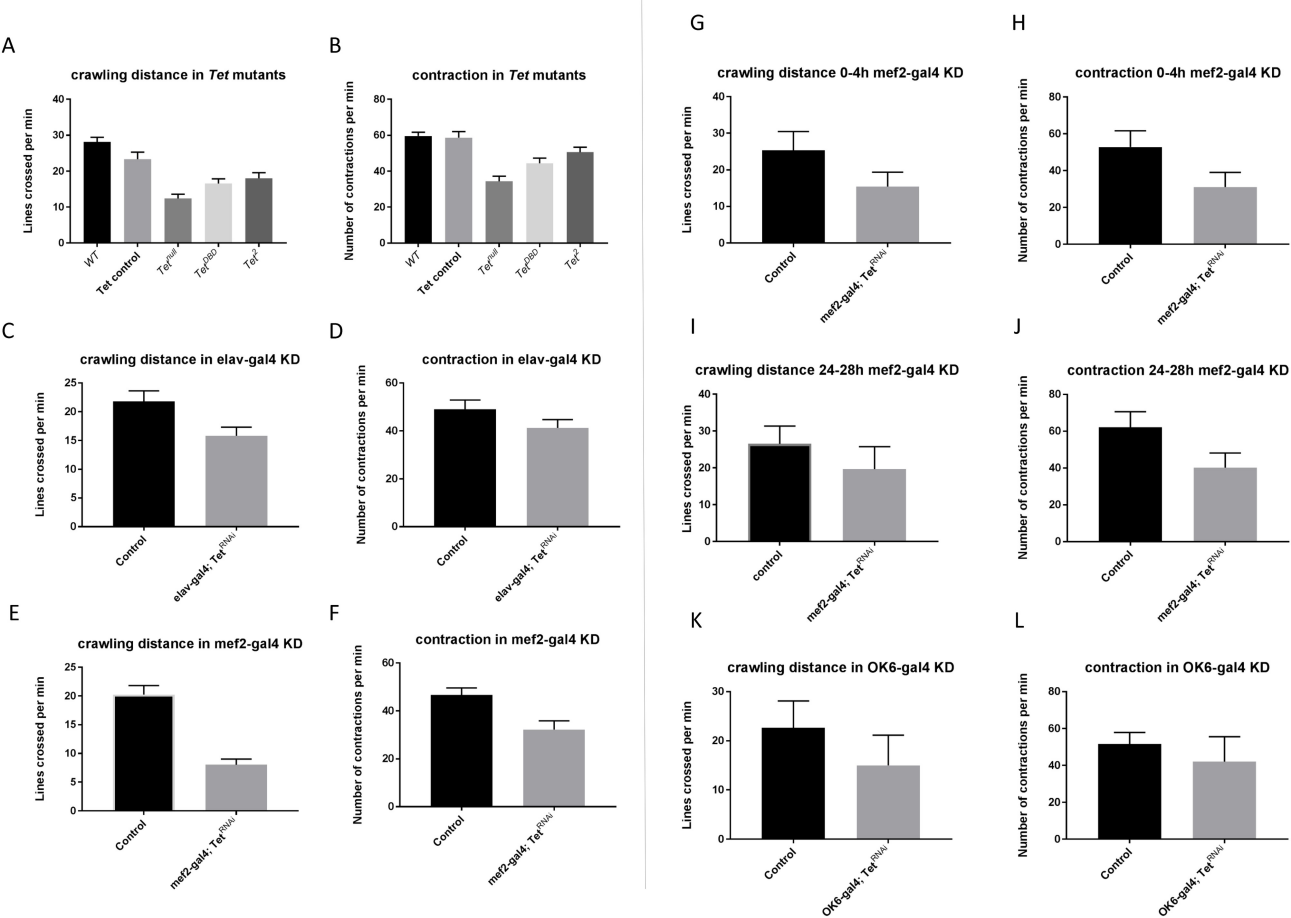
Tet requirement in larval locomotion. (A and B) Locomotion and contractions are significantly affected in *Tet* mutants. Note that *Tet*^*null*^ shows a stronger effect than the alleles that retain some Tet function (A, *p* < 0.0001; B, *p* < 0.001, for all three mutant alleles). WT, w^118^ larvae, Tet control, parental stock containing piggyback transposon PBac{WH}f05022 (C and D) Tet KD by the *mef2-gal4* driver results in strong reduction of locomotion and contractions (C, *p* < 0.0001; D, *p* < 0.01). (E and F) Tet KD by the *elav-gal4* driver results in moderate but significant reduction of locomotion and contractions (E and F, *p* < 0.0001). (G and H) Conditional Tet KD by the mef2-gal4 driver during embryogenesis affects locomotion and contractions significantly (G and F, *p* < 0.0001), but the difference in locomotion and contractions is alleviated when the KD is induced in first instar larvae (I and J) (I, *p* = 0.053; J, *p* < 0.01). (K and L) Tet KD by the *OK6-gal4* motorneuron driver did not affect the larval locomotion and contractions significantly (K, *p* = 0.07; D, *p* = 0.1, for all mutant alleles).

We examined the movements of cohorts of larvae in 1 minute movies, and quantified total distance traveled and crawling speed and observed that *Tet* mutants displayed a loss of motility and contractions. *Tet*^*null*^ larvae showed >50% reduction in body wall contractions and crawling speed (Fig 3A and 3B). The partial loss of function alleles *Tet*^*DBD*^ (62%) and *Tet*^*2*^ (68%) exhibited clear, but less severe defects in both aspects of locomotion (Fig 3A and 3B). The differences were statistically highly significant leading to the conclusion that Tet is required for normal locomotion.

We further investigated if *Tet* function is required in neurons or muscles for normal locomotion. We performed the locomotion assays on third instar larvae in which Tet was depleted either in muscles (mef2-gal4; Tet^RNAi^), in neurons (elav-gal4; Tet^RNAi^), or in a motor neuron subset (OK6-gal4; Tet^RNAi^). Mef2-gal4 driven Tet KD larvae showed the most severe defect similar to that observed in *Tet*^*null*^ animals (Fig 3E and 3F). Elav-gal4 Tet KD displayed a less severe, but significant defect (Fig 3C and 3D), whereas OK6-gal4 KD did not alter the locomotion behavior (Fig 3K and 3L). These results indicate that Tet function is required both in neurons and muscles but not in motor neurons.

Next, we examined the temporal requirement of Tet in locomotion by using the temperature-sensitive *GAL80*^*ts*^ system [23]. GAL80 suppresses the activity of the transcriptional activator GAL4, preventing expression of genes controlled by an UAS promoter, in this case, *UAS-Tet*^*RNAi*^. Raising the temperature to restrictive levels inactivates GAL80^ts^ and permits expression of the *Tet*^*RNAi*^. We used this system to inactivate *Tet* during different developmental stages.

We observed a significant defect in locomotion when embryos were deprived of Tet function during embryogenesis. In mef2-gal4 induced knockdown embryos reared at the restrictive temperature 0–4 h after egg lay (AEL), significant reductions of both contractility and overall motility were observed (Fig 3G and 3H). However, if we reduced Tet function later, when animals are between 24 h and 28 h old (first instar), the variance of the measurement became much greater, so that the defects became insignificant (Fig 3I and 3J). This result indicates that Tet is required during embryogenesis for larval locomotion, but is dispensable for locomotion in 1^st^ instar larvae.

To gain functional insight into the basis of this defect, we tested for a possible role of Tet in synaptic structures by analyzing the neuromuscular junctions (NMJs) of motor neurons terminating on the somatic musculature of third instar larvae. No significant changes in the number of NMJ boutons or their morphology were observed in *Tet*^*null*^ mutants (S3B and S3C Fig).

### *Tet* controls the development of neurons essential for the circadian rhythm

We were interested in determining if Tet was also required in additional neuronal functions. The circadian rhythm sets the day–night clock in most organisms regulating many physiological processes and is well studied in Drosophila. We decided to investigate if Tet is required for circadian behavior. The rhythm of locomotion is controlled by about 150 clock neurons in the central brain, organized into distinct subgroups. The *timeless* (*tim*) gene, expressed in all clock neurons, is one of the major factors controlling the circadian rhythm [24]. Under light/dark (LD) cycles, fruit flies exhibit bimodal activity with morning and evening peaks. Small ventral lateral neurons (s-LNvs) expressing the pigment-dispersing factor (PDF) are necessary and sufficient for morning activity [25] [26].

**Fig 4:**
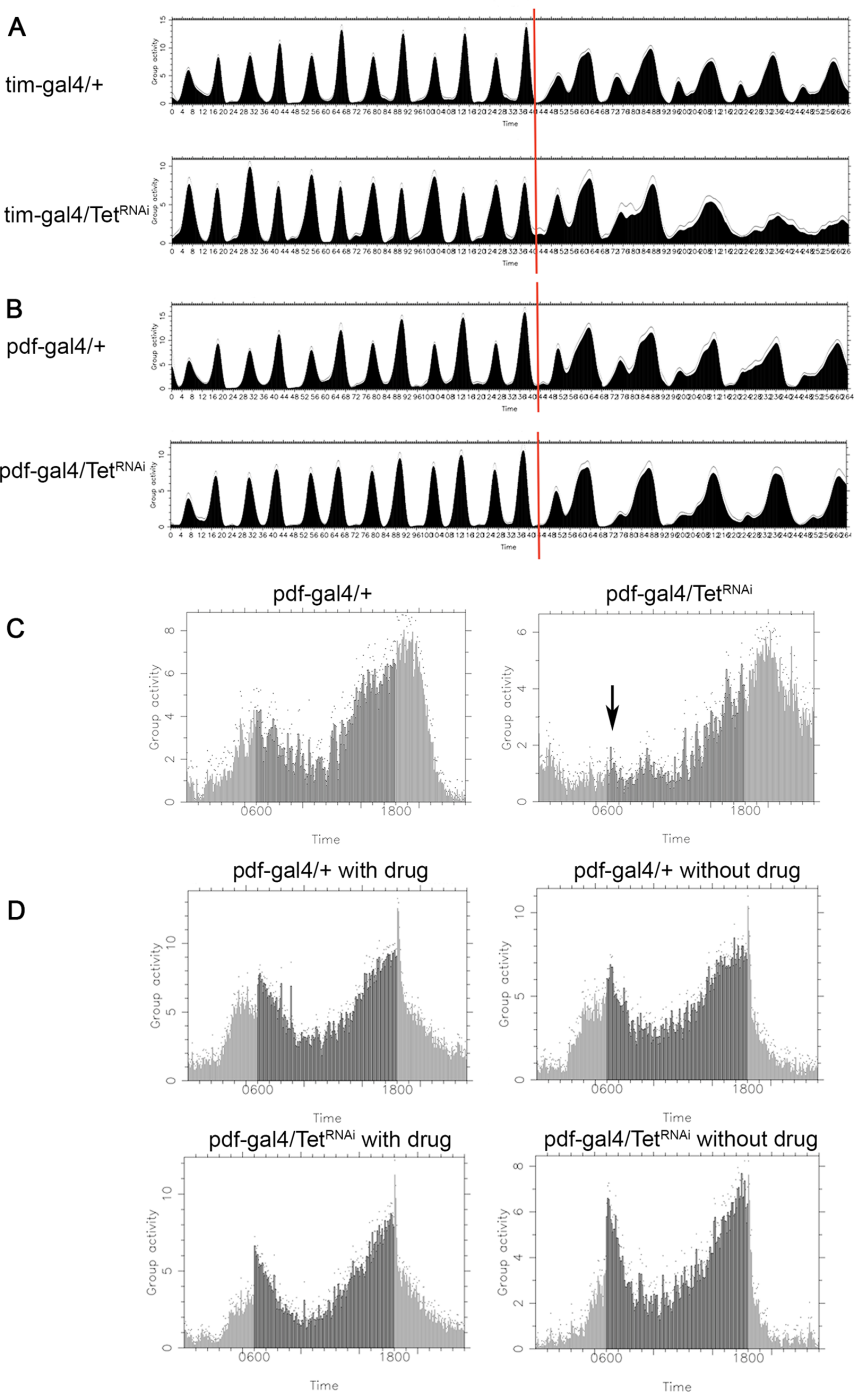
Tet and the circadian rhythm. (A and B) Actograms: Depletion of Tet with the *tim-gal4* driver alters the bimodal pattern of the activity peaks after two days of DD. The morning activity peak is lost on day 2 of DD when Tet is depleted by *pdf-gal4*. (C) Eduction graph of average activities in DD shows the reduction of the morning peak (black arrow) if Tet is knocked-down throughout the expression period of *pdf-gal4*. (D) No effect on the morning peak is observed when Tet is depleted only in the adult stage (pdf-GS-gal4/Tet^RNAi^ with drug) (A and B) Light-Dark training, left of the red line, Dark-Dark behavior, on the right.

To evaluate if Tet functions in these processes, we examined adult behaviors in flies with cell-specific KD of Tet. 3–5 d old males were trained across 12h Light-Dark (LD) cycles for 6 days, followed by constant darkness (DD). Control flies displayed two activity peaks, one before lights-on (morning anticipation), and the other before lights-off (evening anticipation), which were retained for 5 days under DD. When Tet was depleted under tim-gal4 control in all clock neurons and glia cells (tim-gal4, Tet^RNAi^*)*, the bimodal pattern was lost as the morning peaks were missing in Tet KD animals 2 days after switching to DD (Fig 4A). The morning peak is controlled by the PDF-positive s-LNvs neurons (PDF neurons). Therefore, we also examined animals in which Tet was depleted specifically in the PDF neurons (pdf-gal4, Tet^RNAi^) and found that in these animals the morning anticipation was also disrupted (Fig 4B and 4C), while the total activities were not changed (S4A–S4C Fig).

We also investigated the temporal requirement of Tet in PDF neurons. The drug induced gene switch (GS) system provides temporal control of GAL4 [27]. When we controlled KD of *Tet* using the pdf-GS-gal4 driver in adult males, no difference in the morning anticipation was observed between Tet KD and WT (Fig 4D).

**Fig 5:**
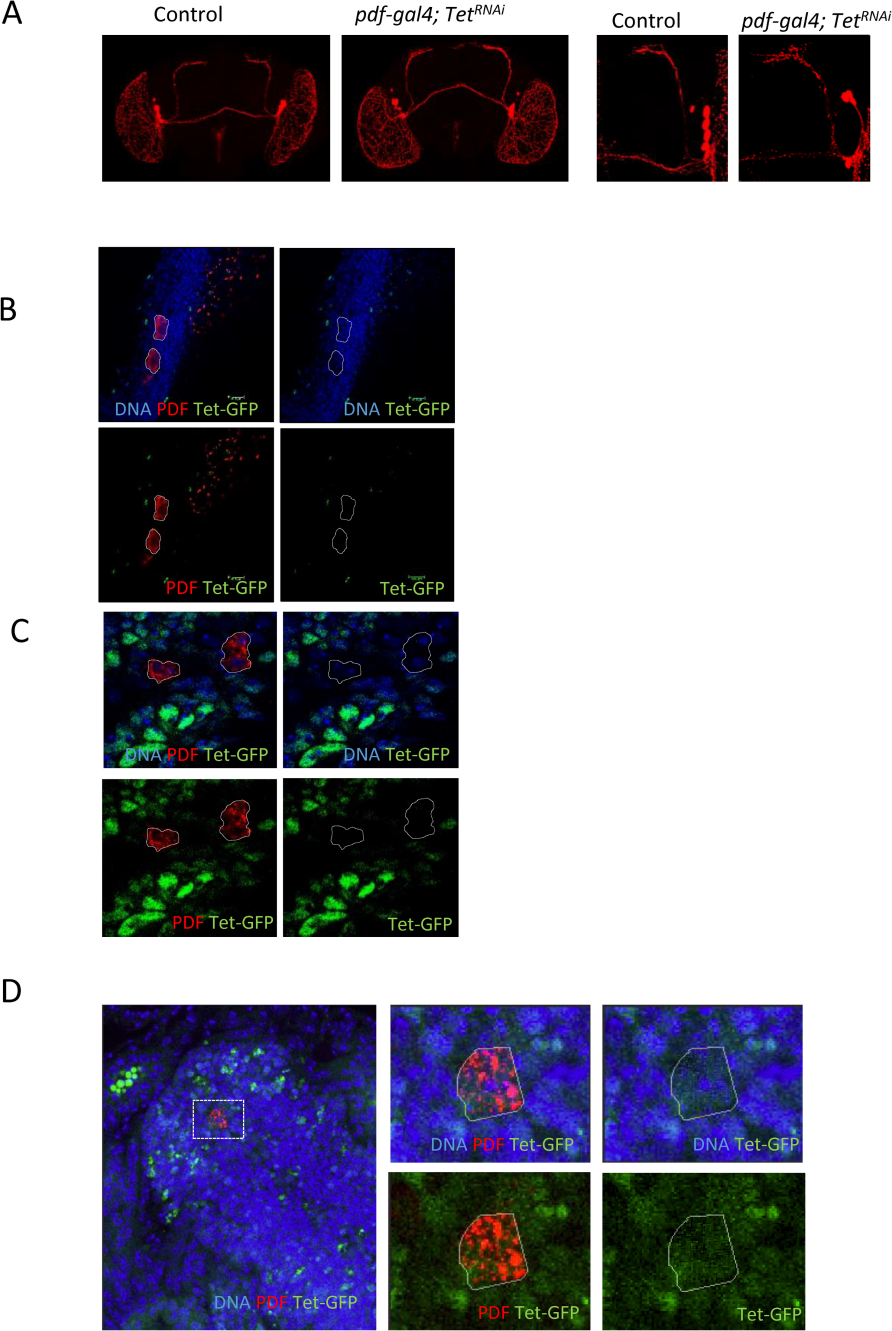
Tet expression in PDF neurons. (A) *Tet KD* by *pdf-gal4* does not affect organization of PDF neurons in adult brain. Brains are stained by anti-PDF antibody. (B) Tet-GFP is not expressed in PDF neurons in adult males or, (C) in third instar larval brains. (D) Tet-GFP protein is observed in the cytoplasm of PDF positive neurons in the embryonic central nervous system (stage 16).

We next investigated if we could detect any abnormalities in the pdf-positive neural network by staining adult brains with anti-PDF antibody of Tet KD flies. The PDF network looked indistinguishable in Tet KD and WT adult brains (Fig 5A). The expression of the PDF marker is normal in pdf-gal4 driven Tet KD brains, and the PDF neurons extended normal axonal and dendritic projections. This rules out the possibility that Tet affects the circadian rhythm through impairing the gross anatomy of pdf neurons. To further address when Tet might function in PDF neurons, we examined Tet expression. We found that in embryonic stage 16, Tet-GFP is observed in the cytoplasm of PDF neurons (Fig 5D), but it is absent in 3^rd^ instar larval or adult stages (Fig 5B and 5C). Our results show that Tet does not directly control the circadian rhythm, rather, that it controls the development of precursors into functional PDF neurons.

### Tet controls ovary development during embryogenesis

Gametogenesis in Drosophila starts in the early embryo when the primitive ovaries form on each side of the gut. During larval development the ovaries increase in volume about 50 fold. The groups of cells making up the larval ovary become subdivided into ~16 ovarioles by the migration of mesodermal cells. At the anterior end of an ovariole are the terminal filament cells, adjacent to the germ line stem cells. The ovarioles contain a series of maturing egg chambers from the youngest, to the mature eggs at the posterior end.

**Fig 6:**
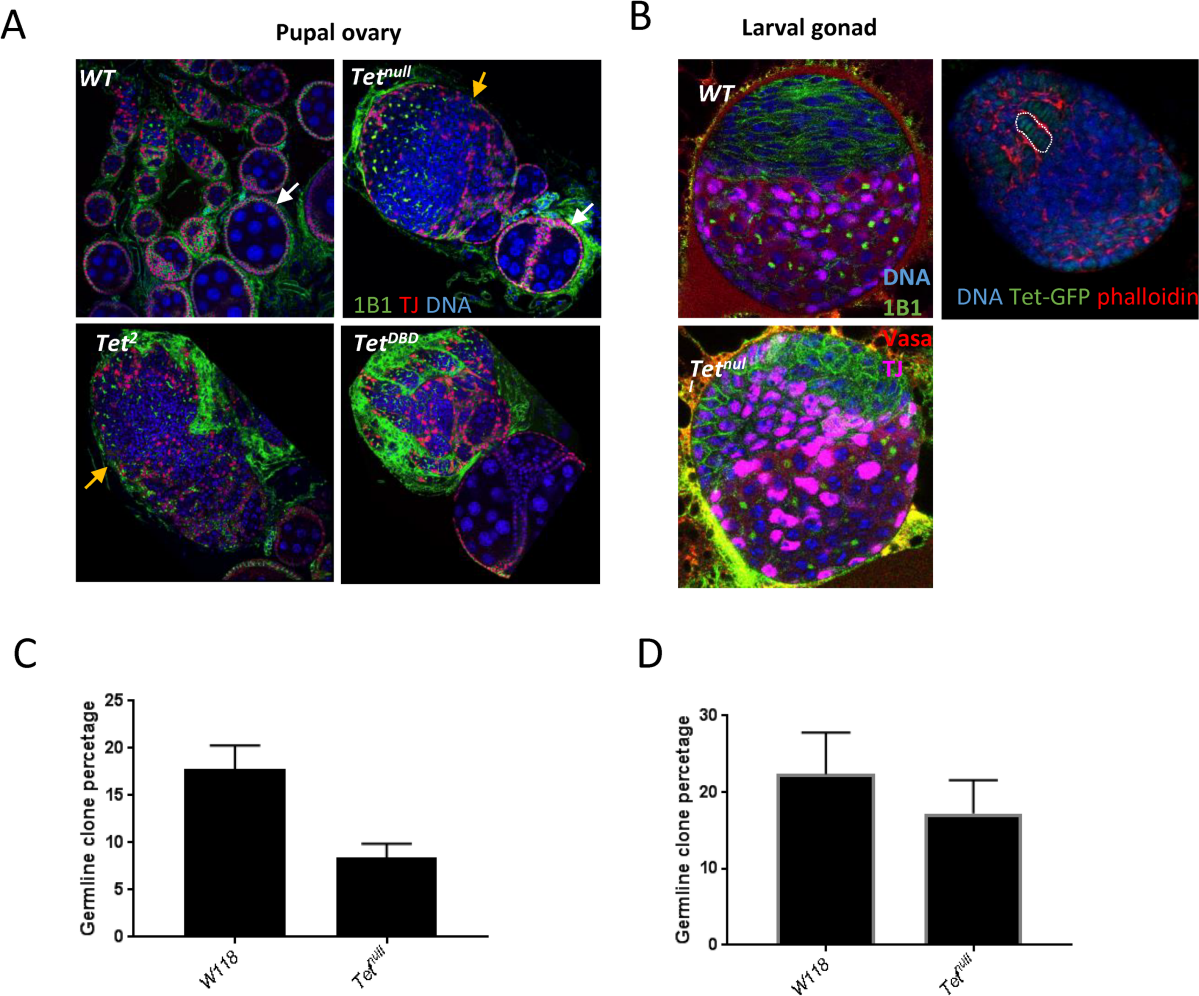
Tet controls ovarian development. (A) The ovarioles are clearly separated and contain up to stage 8 egg chambers (white arrows). *Tet* mutant ovaries fail to separate their ovarioles, but do contain some disorganized egg chambers (orange arrows). The *Tet*^*null*^ ovaries does not show the clear separation of the somatic (TJ, red) and germline tissues. 1B1 in green outlines the somatic cells and fusome. (B) Left side, 3^rd^ instar larval gonad: *Tet*^*null*^ gonad display the defect in cell organization. TJ (purple) labels the somatic cells and Vasa (red) labels the germline cells. Right side, Tet-GFP is observed in terminal filament cells in WT late 3^rd^ instar larval ovaries. Red, filamentous actin, most highly expressed around the terminal filament cells. The germline clones induced in 0-24h embryogenesis showed a strong reduction ((*p* < 0.0001) (C), but not the ones induced at 3^rd^ instar larval stage ((*p* = 0.11) (D). PGC, primordial stem cells; GSC, germ line stem cells.

We examined the requirement for Tet in ovaries by characterizing the *Tet* phenotype. As *Tet*^*null*^ animals die as pharate adults, we examined WT and mutant ovaries dissected from larvae and pharate adults. The *Tet*^*null*^ larval ovaries contain all the cell types present in the WT ovary, however the organization is strongly disturbed (Fig 6B). Late larval WT ovaries show subdivision into ovarioles with the stacked terminal filament cells visible (Fig 6B). By pharate adult stage, the ovaries are similar to those of virgin females with separated ovarioles containing egg chambers that have developed to about stage 8 (Fig 6A). In *Tet*^*null*^ mutants, the ovarioles are not separated and no early egg chamber stages can be discerned. Moreover, the somatic portions of the ovary, such as stacked terminal filament cells cannot be clearly identified. Nevertheless, at the posterior end of the ovary some egg chambers of about stage 8, as judged from their organization and size of their nuclei, can be identified (Fig 6A). *Tet*^*DBD*^ and *Tet*^*2*^ pupal ovaries displayed a similar phenotype with incomplete separation of ovarioles, but the phenotype is less severe. The organization of the ovary and subdivision into ovarioles while incomplete, can be detected (Fig 6A). The phenotypes observed in the larval and late pupal mutant ovaries indicate that the requirement for Tet in ovary morphogenesis occurs before the larval stages.

Next, we investigated where Tet is expressed in fly ovaries. Using the *Tet-GFP* line, we first examined the adult ovary and found no expression of Tet in either somatic or germline cells including germline stem cells. We also examined the earlier stages for Tet expression. We did not see specific expression in pole cells or the embryonic gonad in the Tet-GFP line, but we did observe weak nuclear expression in the terminal filament cells in the late 3^rd^ instar larval gonads, which could at least in part explain the observed disruption of organization of the ovarioles in *Tet*^*null*^ pupal ovaries (Fig 6B).

To further investigate the requirement of *Tet* in ovary development, we induced clones by flippase recognition target (FRT)-mediated site-specific mitotic recombination [28]. Phenotypes were analyzed 10 or 20 days after clone induction (ACI). We first induced clones in 3^rd^ instar larvae and found that both germ line and somatic (follicle cells) WT and *Tet*^*null*^ clones were similar in numbers and morphology (Fig 6D, S5D Fig). Next, we induced clones in 0-24h h embryos, which will only result in germ line clones. We detected reduced numbers of Tet germ line clones; in WT 17.8% of ovarioles contained clones compared to 8.4% of ovarioles that contained *Tet*^*null*^ clones (Fig 6C). However, the *Tet* clones did not show any visible phenotypes and the egg chambers looked similar to the WT egg chambers. This reduction in clones indicated that there is a requirement for Tet function in ovary development during embryogenesis.

## Discussion

### Tet functions in neurons and muscle precursors

In our previous work, we mapped the transcriptome-wide 5hmrc landscape in S2 cells and found that transcripts encoded by genes involved in neuronal differentiation contained the most 5hmrC peaks. These genes also represent a significant percentage (26%, P<10^43^) of the Tet-regulated transcripts as determined by sequencing of RNAs isolated from normal and Tet depleted S2 cells. We further reported that Tet was an essential gene and that *Tet*^*null*^ larval brains showed a small, but significant defect in development [4]. Our further experiments into the brain phenotype (induction of *Tet*^*null*^ clones and Tet KD with brain-specific drivers) showed that the observed reduction of the medulla of larval brains was most likely due to a developmental delay.

The results obtained in S2 cells are corroborated and extended by the *in vivo* data we report here. We found that Drosophila Tet protein is expressed most highly in the central nervous system, especially the brain, starting at germ band extension and continuing throughout development. This agrees well with observations that the 5hmrC mark is highest in larval brains [4]. Although we did not observe lethality after depletion of Tet from neuronal cells, we found that Tet was required for normal circadian rhythm and especially early in development in pdf neurons for the morning peak of the rhythm.

Tet protein is present in somatic muscle precursors in the embryo, where it appears to have an essential function. 3^rd^ instar *Tet*^*null*^ larvae exhibit normal morphology but severe motility defects and newly eclosed hypomorphic adult flies have uncoordinated leg and wing movements. These defects could either derive from Tet loss of function in the postmitotic neurons, or from Tet loss in muscle precursor cells. Our results show that Tet expression in neurons and muscle precursor cells is highest during embryonic development and is also required in both tissues during embryogenesis for normal larval locomotion. Further, this time window of Tet activity overlaps with the period when Tet is required in PDF expressing neurons to assure normal bimodal circadian rhythms. These results are consistent with Tet having an essential function in neuronal and muscle development during embryogenesis. Consistent with the notion that the Tet-dependent deposition of 5hmrC serves as an epigenetic mark, the absence of Tet function during embryogenesis results in phenotypes which become manifest at later developmental stages (e.g., larval or adult) whereas the absence of Tet function during late larval stages has little or no affect upon the processes investigated in this study.

In vertebrates Tet levels are especially high in ESCs and in neurons [29] [30] [31] and requirement of Tet proteins in neurogenesis has been documented in several species. In Xenopus Tet3 is strongly expressed in embryonic developing neuronal tissues and depletion of Tet3 using Morpholino antisense Oligo-nucleotides, resulted in abnormalities in the development of these tissues and in death [32, 33]. Further, mouse Tet3 KO ES cells, while normal in self renewal, fail to differentiate into viable neural precursor cells [34]. Also, Tet1 KO mice develop normally with no visible morphological or growth defects. However, when 4 months old WT and Tet1 deficient mice were tested in the Morris water maze, a deficit in learning and short term memory was detected in the mutant animals [35]. In the literature these phenotypes have been interpreted as resulting from a lack of 5hmC in DNA, but it seems possible that 5hmrC may also be involved in these neuronal processes. Tet proteins have also been linked to muscle development and function in vertebrates, but their direct involvement has not been clearly documented so far [36, 37].

*Tet*^*null*^ animals die in late pupal stages and the lethality can be separated from the larval locomotion phenotype in that Tet appears to be required in mesodermal cells throughout embryonic and larval development for viability, correlating with the expression of the *Tet* gene. It is therefore possible that Tet function may be essential in additional cells and tissues in which it is expressed, such as adult tissues derived from the Tet positive larval disc cells.

### Requirement of Tet in the ovary

We demonstrated that Tet is required for normal oogenesis in the embryonic stages. In our clonal analysis we observed a reduction in *Tet*^*null*^ egg chambers when clones were induced during embryogenesis, but induction in 3^rd^ instar larvae did not reduce the number of clones in the germline or the soma. These results are consistent with *Tet* functioning in germline cells during embryogenesis and early larval stages. In addition, we found that complete loss of *Tet* in Drosophila results in a strong phenotype in larval and pupal ovaries: the ovaries are disorganized and mesodermal cells fail to migrate to form ovarioles. Together with the observed expression of Tet in somatically derived terminal filament cells, the phenotypes suggest that Tet is required the somatic cells of the developing ovary.

Tet was shown previously to be both expressed and required in the germline in early oogenesis [38]. We did not observe expression of Tet during these stages. Therefore we investigated if there was any maternal requirement for Tet function during oogenesis. To this end we induced *Tet*^*null*^ clones by the OvoD technique, which allows creation of homozygous mutant embryos from mutant eggs. In our experiments the homozygous *Tet*^*null*^ embryos derived from *Tet*^*null*^ stem cells did not show any enhanced phenotype compared to the embryos derived from *Tetnull/+* stem cells. Consistent with the lack of Tet expression in germline cells during oogenesis, our result further indicates that maternal Tet is not required for normal development of the offspring.

Our results on the requirement for Tet during oogenesis differ significantly from those previously published by Zhang et al. [38] We could not confirm their reported presence of the Tet protein during oogenesis and in 0-2h embryos. Further, when we induced clones in third instar larvae we did not observe any effect on early germ cell differentiation. The differences in expression pattern may be due to the fact that Zhang et al. used an anti-Tet antibody, that may not have recognized the Tet protein specifically, while we demonstrated the absence of Tet expression in adult ovaries and 0–2 hour embryos using the Tet-GFP reporter line both by fluorescent imaging and western blot (S1B Fig).

### The Tet proteins

Drosophila has only one *Tet* gene, encoding several alternatively spliced isoforms from two distinct promoters. Drosophila Tet-L most closely resembles mammalian TET1 and 3, while Tet-S is similar to TET2. Both DNA binding and catalytic domains of Drosophila Tet are about 50% homologous to that of TET1/3, and the specific amino acids within the catalytic domain that are responsible for methylcytosine binding are identical in Drosophila and vertebrates [20]. Homozygous or hemizygous *Tet*^*DBD*^ and *Tet*^*2*^ animals die, like *Tet*^*null*^, at the late pupal stage while *Tet*^*DBD*^/*Tet*^*2*^ show strong complementation (Table 1). But the trans-heterozygotes adults live up to two weeks and have normal looking ovaries (S5C Fig), and females are sterile. This result indicates that both forms of Tet have essential functions.

Mammals have three Tet genes: Tet 1 and Tet3 encode proteins with both a DNA-binding and a catalytic domain, and Tet2 that lacks the DNA-binding domain. Xenopus however, has only two Tet genes, encoding the two basic forms of Tet, one form containing both domains and one from containing only the catalytic domain. Drosophila has only one Tet gene, encoding the two basic forms of Tet. Vertebrate genes, while showing specific expression patterns are partially redundant. For instance, Tet1 and Tet2 KO mice survive, but the majority of double KO mice die within two days postnatally [39], and Tet3 KO mice die one day postnatally [8]. This suggests that Tet3 has a distinct essential function which could be accounted for through its demonstrated role in RNA hydroxymethylation.

The catalytic domains of all three Tet enzymes as well as full-length Tet3 have been shown to induce the formation of 5-hmrC in RNA in mouse and human cells [15]. Of the three Tet proteins, human TET3 is structurally most similar to Drosophila [20] and TET overexpression experiments in a human cell line suggests that hydroxmethylcytosine formation in RNA may be controlled solely by TET3 [15]. Further, *Tet3* expression is reported to be particularly high in the developing mouse brain [12]. Because both the Tet-L (similar to Tet3) and Tet-S (similar to Tet-2) are essential in flies where 5hmC in DNA is lacking, Tet2 may be functioning with Tet3 in controlling 5hmrC also in vertebrates. But it is also possible that Tet proteins have additional, non-catalytic, essential functions in flies that are conserved in vertebrates.

In studying the expression and distribution of Tet-GFP in embryos and larvae we find that the protein is found primarily in the nucleus, but can also be detected exclusively in the cytoplasm, for instance in PDF expressing neurons in the embryo. As Tet-L contains a DNA-binding domain, we hypothesize that its major function is in the nucleus, where it may control the modification of nascent transcripts. It is possible that Tet-S represents the cytoplasmic protein. Independent of the localization of the Tet proteins, they control the modification of specific mRNAs and likely their transport and translation [4].

Together with the documented function of vertebrate proteins in RNA modification, our results invite further investigation of 5hmrC in vertebrate RNA, particularly in neuronal and muscle development and function. It is well possible that phenotypes described in mouse mutants, or symptoms associated with Tet in humans, could be due to abnormal processing of specific mRNAs, and not exclusively in response to disruption in hydroxymethylation of Cytosine in DNA.

## Material and methods

### Fly lines

The deletion alleles *Tet*^*DBD*^ and *Tet*^*2*^ were generated by FRT site-specific recombination between two PBac insertions, obtained from Exelixis at Harvard Medical School (Fig 1). *Tet*^*DBD*^ was recombined using *PBac{WH}f0306* and *P{XP}d00815*. *Tet*^*2*^ was recombined using *PBac{WH}f05022* and *P{XP}d00815*. The mutant chromosomes are kept over balancer chromosomes *TM3* or *TM6*. The Tet RNAi lines (#v110459, #v102273, #v20798, #v36178) were obtained from the Vienna *Drosophila* RNAi Center (VDRC). We found that #v102273 showed the most efficient KD and therefore this line, called TetRNAi in this work, was used for all experiments. The *mef2-gal4*, *da-gal4*, *tub-gal4*, *act5-gal4*, *elva-gal4*, *pdf-gal4*, *tim-gal4*, *UAS-dcr2*, and deficiency stocks were obtained from the Bloomington Stock Center. For RNAi experiments, flies were kept at 25°C. For temperature shift experiments, we used 25°C as permissive temperature and 29°C as restrictive temperature.

### Clonal analysis

The FLP/FRP site-specific recombination system was used to generate mutant clones under the control of a heat-shock promoter [28]. The fly stocks used to induce ovoD clones and have been previously described [40, 41]. To induce clones, 0-24h embryos and third instar larvae of the appropriate genotype were exposed to 38°C for 90 minutes. Ovaries were dissected, fixed stained at 5–10 days after eclosion. To count and characterize clones, more than 150 ovarioles of each genotype were analyzed on a Zeiss Axioplan-2 microscope.

### Antibodies and microscopy

Embryos were dechorionated in 50% bleach, fixed in formaldehyde-heptane and devitellinized with methanol. Embryos were permeabilized and blocked in PBST and 5% goat serum for 1 hours. Embryos were incubated in primary antibodies overnight at 4°C overnight, and in secondary antibodies for 2hrs at room temperature. The following primary antibodies were used: rat-anti-Elav (1:250, DSHB), mouse-anti-Prospero (1:20, DSHB), rabbit anti-Mef2 obtained from H. Nguyen, University of Erlangen-Nuremberg, Nurenberg, Germany (1:1000) [42], rabbit anti-ß3 tubulin from R. Renkawitz-Pohl, Philipps-Universität Marburg, Marburg, Germany (1:1000) [43], and rabbit anti-GFP (1:1000, Invitrogen). Alexa Fluor-488, 568, 647 secondary antibodies were from Molecular Probes and used at 1:500.

For staining of larval NMJ, 3^rd^ instar wandering larvae were dissected as described in Brent et al 2009, [44, 45]. 3^rd^ instar larvae were dissected in calcium free HL-3 saline and fixed in 4% paraformaldehyde in PBT (PBS + 0.05% Triton X-100). Larvae were then washed briefly in 0.05% PBT for 30 min and incubated overnight at 4 with the following primary antibodies: mouse anti-DLG, (1:100, DSHB); TRITC-conjugated anti-HRP (1:200, Jackson ImmunoResearch). Synaptic boutons and NMJ expansion were quantified with Leica software.

Confocal images were captured using a Leica TSC SP5/8 laser scanning confocal microscope (objectives 40× and 63× oil), analyzed with Leica Microsystems software and further processed using Adobe Photoshop.

### Quantitative RT-PCR

We used RNA from third instar larval brain for quantifying Tet mRNA levels. Total RNA was isolated from 20 brains using the RNeasy Plus Mini kit (Qiagen). Quantitative RT-PCR was performed as described in the manufacturer’s instructions using the SYBR Green Selected kit (Qiagen) and the relative standard curve method. Primers used for RT-PCT are listed in S1 Table. Transcript levels were normalized to those of *Rp49*. *RNA purified from* WT larval brains was used as control, and all data were normalized to the transcript levels in WT (baseline = 1). At least three biological and three technical replicates were performed for each genotype. Statistical significance (*P*-value) was determined using two-tailed Student’s *t*-test.

### Fly assays

Lethal phase analysis: Flies were cultured at 25°C and allowed to lay eggs for 12 hours. For each genotype, 100 embryos were collected and transferred to a sugar agarose plate and each plate was scored for the number of hatched larvae and pupae. Experiments were repeated for 3 times.

Larval locomotion assay: Larval locomotion was assayed as described in Louis et al. 2008 [46] but without odor source. Briefly, single mid-3rd instar larvae were placed on 3% agarose plates over a grid and animal locomotion was recorded by a camera for 1 min from the larva’s first movement. 30 to 100 animals were tested for each genotype. Student’s t-test was applied to evaluate the statistical significance.

Circadian Rhythm assay: Adult male flies (2–5 day old) were used to test locomotor activity rhythms [47]. Flies were entrained under LD (the standard condition of 12 h of light and 12 h dark) for 6 days and released into constant darkness (DD) for at least 5 days at 25°C. FaasX software was used to analyze behavioral data to produce the actogram and eduction graph. Activity counts were collected in 5-min bins, and the data for individual flies were pooled to generate group averages of D2 in DD.

## Supporting information

S1 Fig Tet mRNA and protein expression. (A) High throughput RNA seq; Tet is most highly expressed in 2–12 h embryos and in 3^rd^ instar discs and brains (from flybase). (B) Tet-GFP, similar to the Tet RNA, is detected in 8–12 h embryos and larval brains (Western blot reacted with anti-GFP antibody).
(TIF)

S2 Fig Tet KD. (A) Tet RNA depletion in different Tet-RNAi lines using the *da-gal4* driver. 0–12 hour embryos were used for the experiments. (B) Tet-GFP is absent in half of the eye disc when Tet RNAi expression is controlled by the DE-gal4 driver, active on the dorsal side of the eye disc.
(TIF)

S3 Fig Mutant larval size and neuromuscular junctions. (A) The WT and mutant 3^rd^ instar larvae look identical in size. (B and C) The number and morphology of boutons is not changed in *Tet*^*null*^ larvae ((*p* = 0.23).
(TIF)

S4 Fig Period length in *pdf-gal4* KD flies. (A) Average activities are not changed in control and *pdf-gal4* Tet KD adult males ((*p* = 0.43). (B) but period length was prolonged in *pdf-gal4* Tet KD males ((*p* < 0.0001). (C) Period length was not affected when Tet is only depleted in adult stage.
(TIF)

S5 Fig *Tet* mutant ovary. (A) *Mi{MIC}Tet*^*MI03920*^*/Tet*^*null*^ adults show a held-out phenotype and are uncoordinated, before dying 2–3 days after eclosion. (B) The adult ovary phenotype of this mutant shows some separation of ovarioles, but overall the ovary does not look much different than other *Tet* alleles that do not survive well into adulthood (Fig 6). (C) *Tet*^*DBD*^/*Tet*^*2*^ ovary shows no significant difference from control. (D) *Tet*^*null*^ germline white arrow) and somatic clone (red arrow) show no phenotype.
(TIF)

S1 Table PCR primers. (TIF)
