# Supplementary figures and images for: Tet protein function during Drosophila development
Source: PLoS One. 2018 Jan 11;13(1):e0190367. doi: 10.1371/journal.pone.0190367 (PMC5764297; doi:10.1371/journal.pone.0190367)

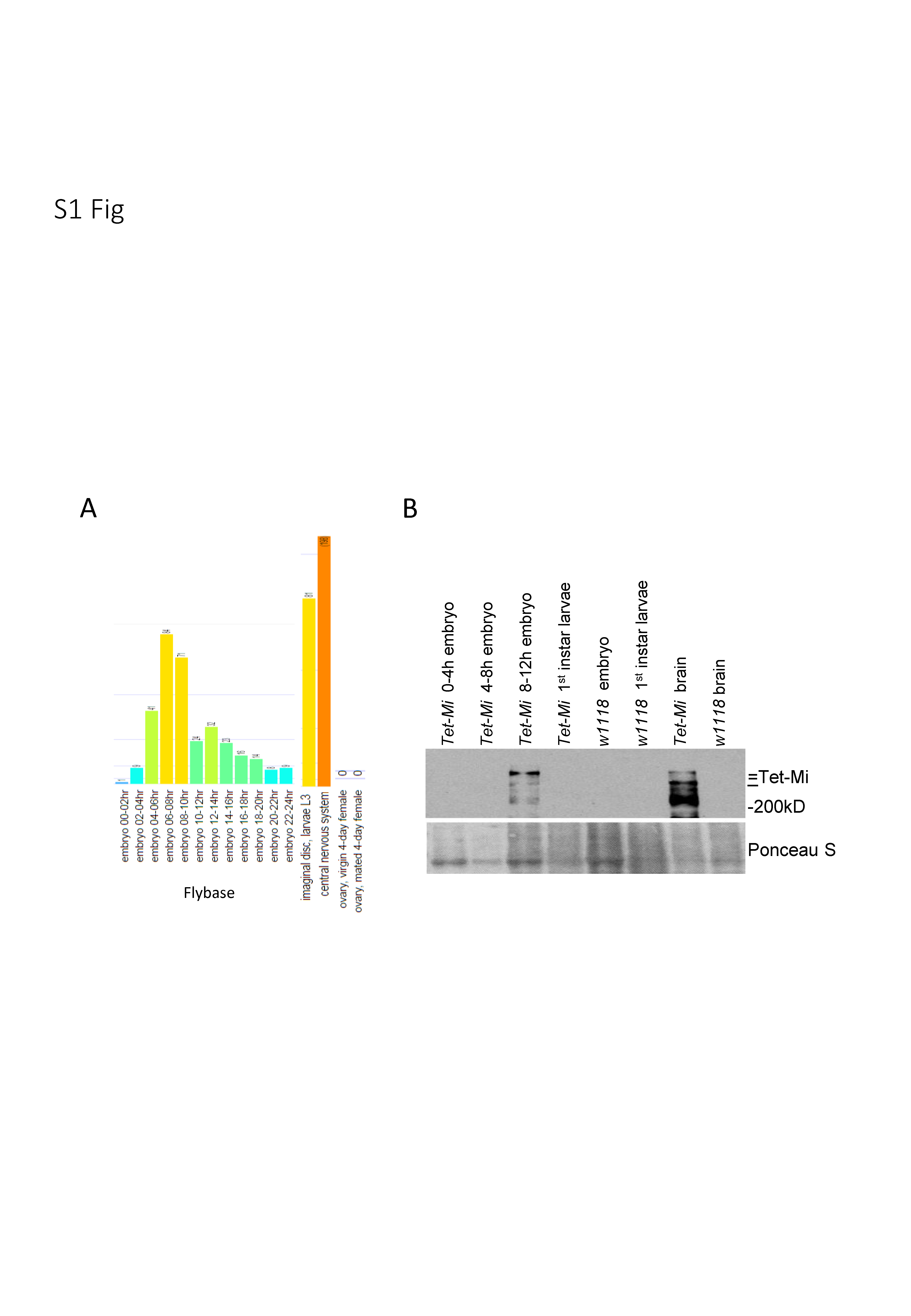

Supplement: S1 Fig — (A) High throughput RNA seq; Tet is most highly expressed in 2–12 h embryos and in 3rd instar discs and brains (from flybase). (B) Tet-GFP, similar to the Tet RNA, is detected in 8–12 h embryos and larval brains (Western blot reacted with anti-GFP antibody). (TIF) [file pone.0190367.s001.tif]

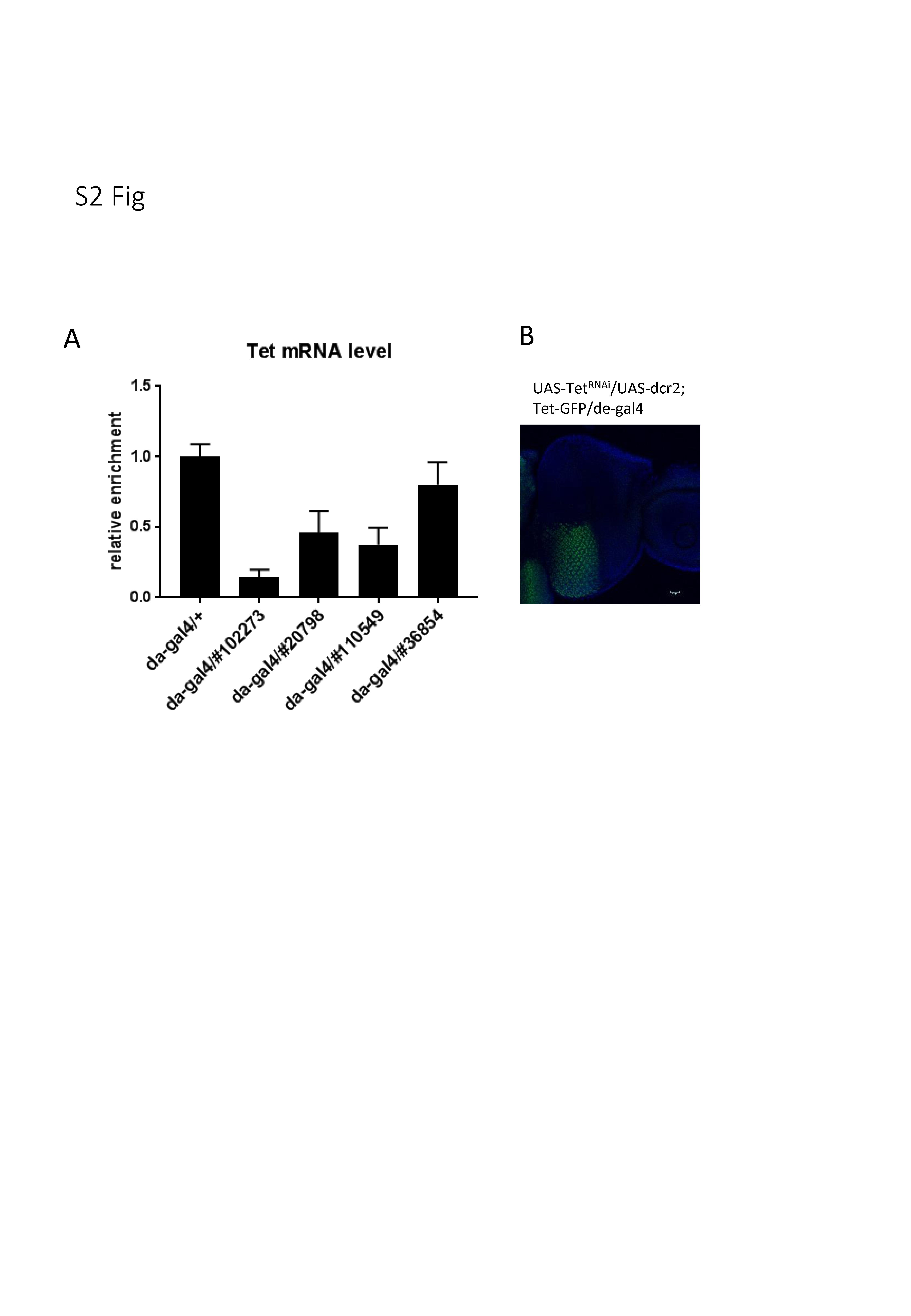

Supplement: S2 Fig — (A) Tet RNA depletion in different Tet-RNAi lines using the da-gal4 driver. 0–12 hour embryos were used for the experiments. (B) Tet-GFP is absent in half of the eye disc when Tet RNAi expression is controlled by the DE-gal4 driver, active on the dorsal side of the eye disc. (TIF) [file pone.0190367.s002.tif]

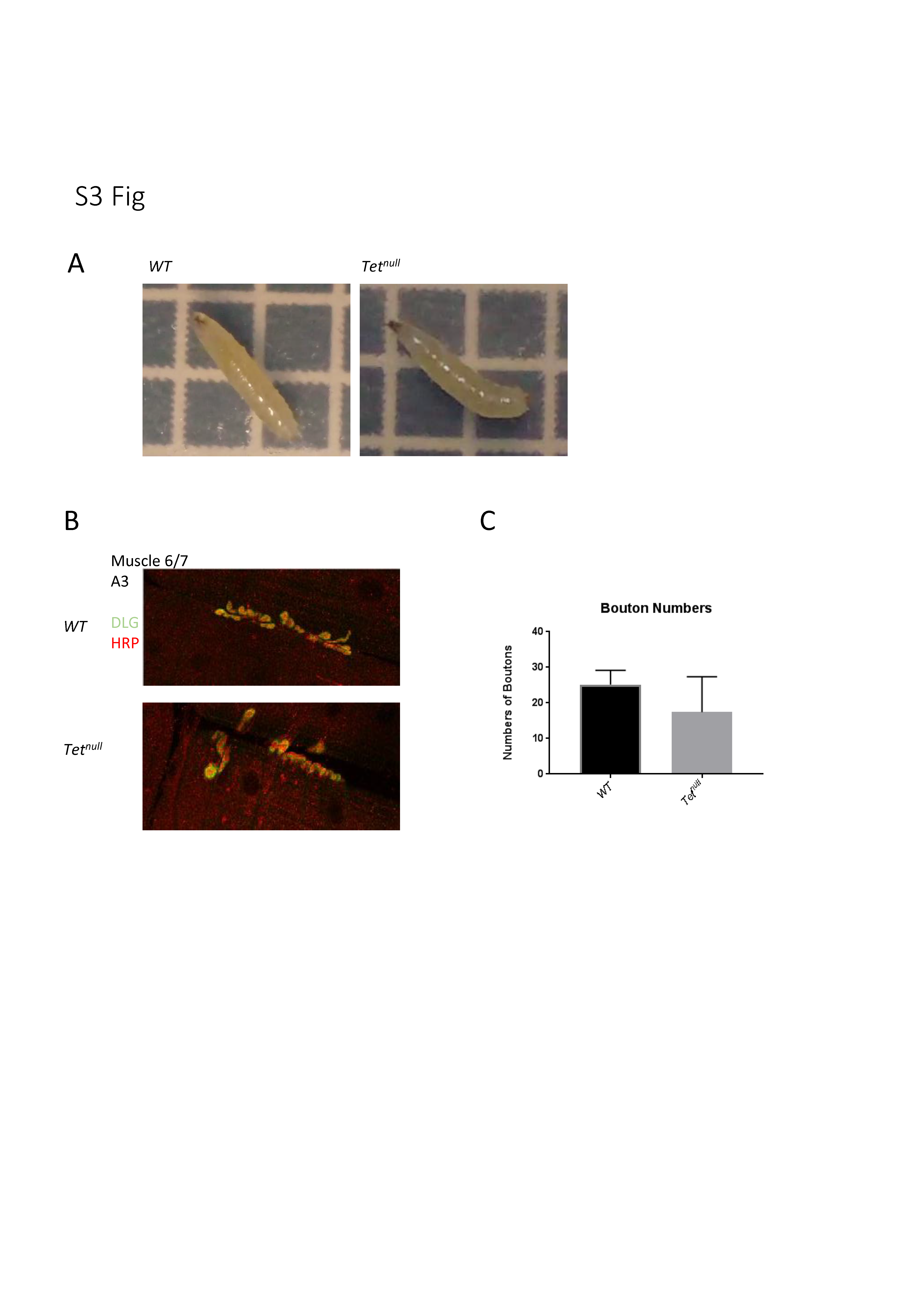

Supplement: S3 Fig — (A) The WT and mutant 3rd instar larvae look identical in size. (B and C) The number and morphology of boutons is not changed in Tetnull larvae ((p = 0.23). (TIF) [file pone.0190367.s003.tif]

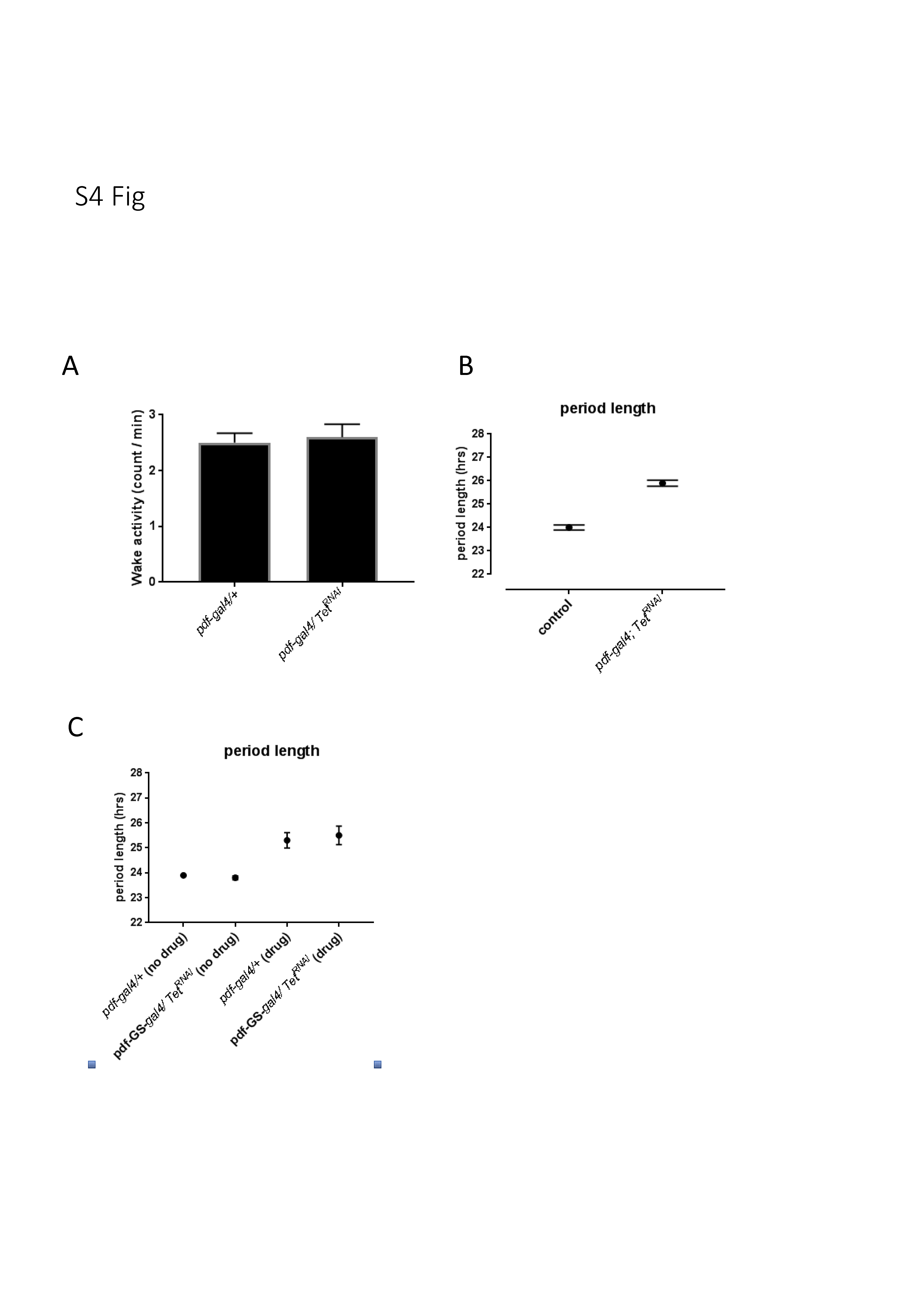

Supplement: S4 Fig — (A) Average activities are not changed in control and pdf-gal4 Tet KD adult males ((p = 0.43). (B) but period length was prolonged in pdf-gal4 Tet KD males ((p < 0.0001). (C) Period length was not affected when Tet is only depleted in adult stage. (TIF) [file pone.0190367.s004.tif]

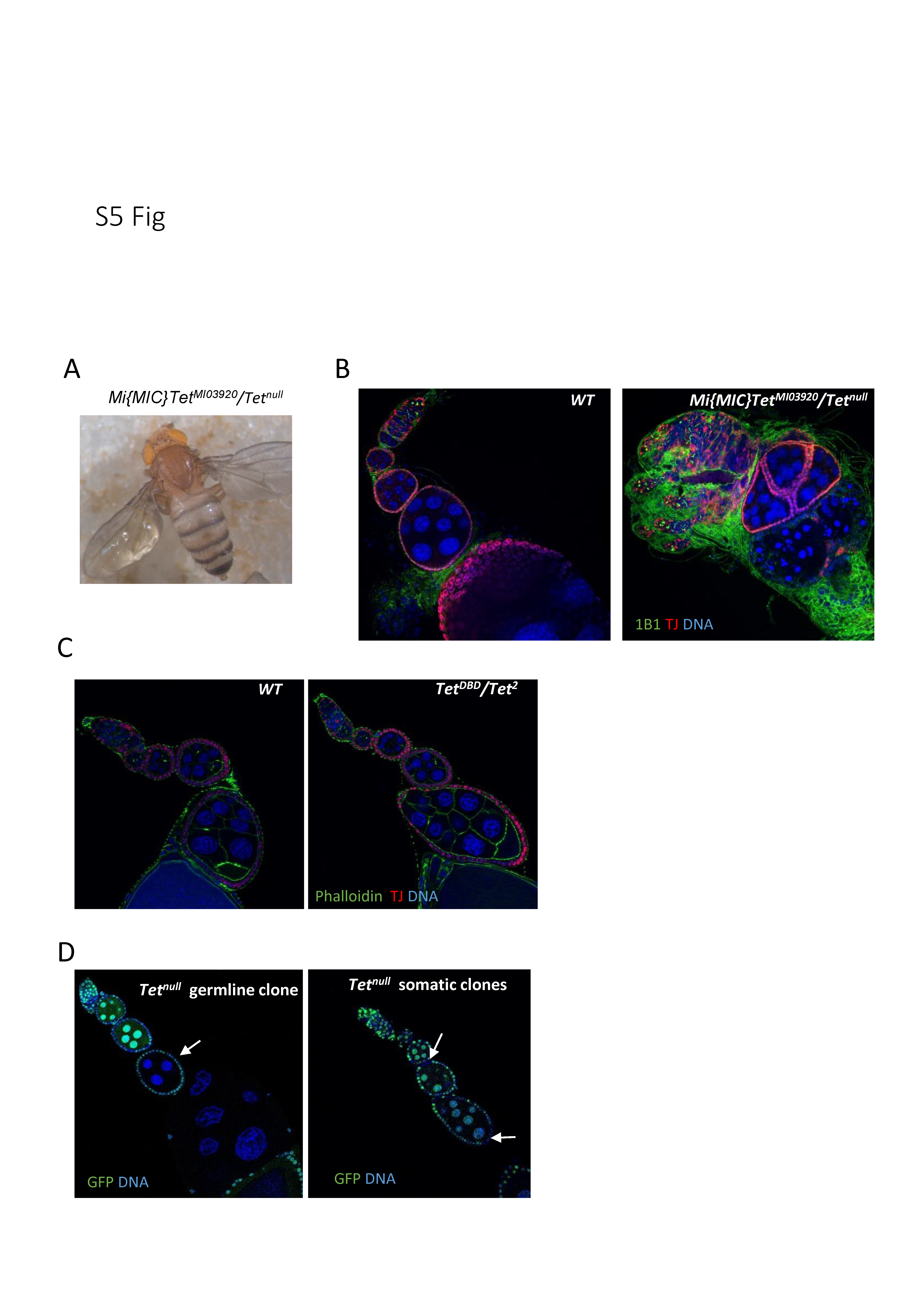

Supplement: S5 Fig — (A) Mi{MIC}TetMI03920/Tetnull adults show a held-out phenotype and are uncoordinated, before dying 2–3 days after eclosion. (B) The adult ovary phenotype of this mutant shows some separation of ovarioles, but overall the ovary does not look much different than other Tet alleles that do not survive well into adulthood (Fig 6). (C) TetDBD/Tet2 ovary shows no significant difference from control. (D) Tetnull germline white arrow) and somatic clone (red arrow) show no phenotype. (TIF) [file pone.0190367.s005.tif]

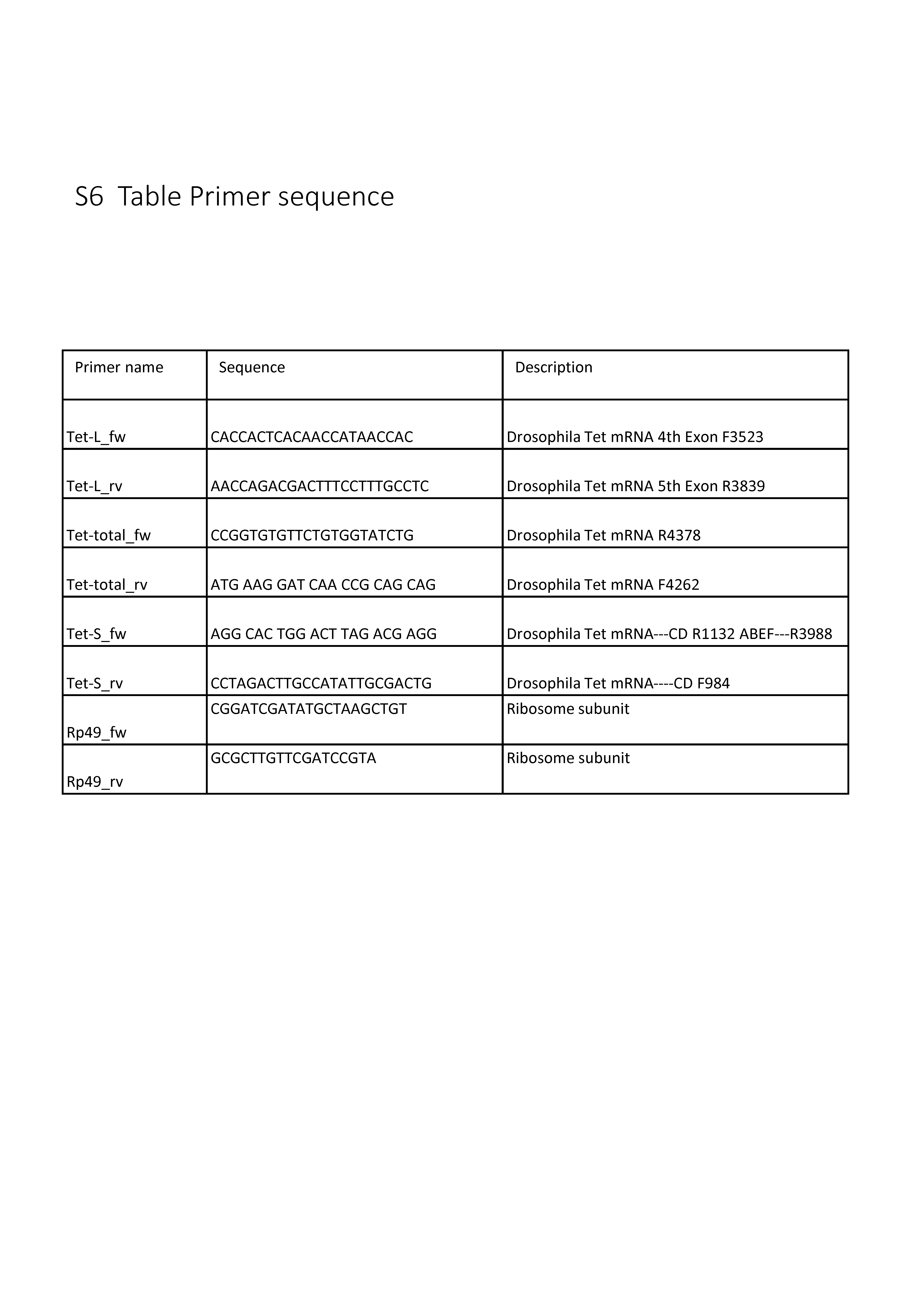

Supplement: S1 Table — (TIF) [file pone.0190367.s006.tif]
